# Cervical Cancer Screening Uptake and Associated Factors among HIV-Positive Women in Ethiopia: A Systematic Review and Meta-Analysis

**DOI:** 10.1155/2020/7071925

**Published:** 2020-08-17

**Authors:** Birye Dessalegn Mekonnen

**Affiliations:** Department of Nursing, Teda Health Science College, P.O. Box: 790, Gondar, Ethiopia

## Abstract

**Background:**

Women living with human immunodeficiency virus (HIV) are more likely to develop an increased risk of invasive cervical cancer. Morbidity and mortality due to cervical cancer could be reduced with early detection through cervical screening. Though uptake of cervical screening was investigated in Ethiopia, inconsistent findings were reported. Therefore, this systematic review and meta-analysis was designed to estimate the pooled prevalence of cervical cancer screening uptake among HIV-positive women and its associated factors in Ethiopia.

**Methods:**

A comprehensive search of PubMed/MEDLINE, Scopus, EMBASE, CINAHL, Google Scholar, Science Direct, and Cochrane Library was conducted. The data were extracted using a standardized data extraction format. Statistical analysis was done using the STATA, version 14, software. The heterogeneity of the studies was assessed using the *I*^2^ test. Funnel plots and Egger's test were used to check publication bias. A random effects model was computed to estimate the pooled prevalence of cervical cancer screening uptake. Moreover, pooled odds ratios with 95% confidence intervals were used to determine the association of identified determinant factors with cervical cancer screening uptake.

**Results:**

A total of 10358 studies were retrieved, and 7 studies were included in the meta-analysis. The pooled prevalence of cervical cancer screening uptake among HIV-positive women in Ethiopia was 18.17% (95% CI : 11.23, 25.10) with exhibited heterogeneity (*I*^2^ = 96.6%; *p* < 0.001). Educational status of women (AOR = 3.50; 95% CI : 1.85, 6.07), knowledge of women on cervical cancer (AOR = 3.26; 95% CI : 2.50, 4.43), and perceived susceptibility (AOR = 3.26; 95% CI : 2.26, 4.26) were significantly associated with cervical cancer screening uptake among HIV-positive women.

**Conclusion:**

The uptake of cervical cancer screening among HIV-positive women in Ethiopia was low. The findings of this study suggest the need to improve the existing national strategies of cervical cancer screening so as to strengthen reproductive health education and promotion, in addition to providing screening services. Furthermore, cervical screening service should be integrated to the routine care and treatment, so that HIV-positive women can get counseling services in every clinical contact.

## 1. Background

In most of the developing countries, both morbidity and mortality due to cervical cancer (CC) are increasing [1–3]. The development of invasive cervical cancer is mainly caused by the human papillomavirus (HPV) infection [4, 5]. Cervical cancer is a global public health concern with highest incidence documented in sub-Saharan Africa ranging from 43.3 to 69.8 per 100,000 women [6]. Recent report from the Information Centre on HPV and Cancer revealed that approximately 29.43 million women aged above 15 years were at risk of CC in Ethiopia [7].

Several studies indicated that women living with human immunodeficiency virus (HIV) have a greater incidence of HPV infection than do the general population [8, 9]. They are also more likely to develop an increased risk of premalignant lesion of the cervix [10, 11]. The literature revealed that immunosuppression with low CD4 counts predisposes women living with HIV infection to high risk for cervical cancer [9, 12–15]. In addition, HIV is associated with several enabling factors for cervical cancer, including multiple sexual partners, early sexual debut, economic status, and smoking [5, 14, 16].

This association of HIV infection and cervical cancer could be more relevant in developing countries including Ethiopia, where access to highly active antiretroviral therapy (HAART) and other services are still existent challenges [17, 18]. According to the World Health Organization (WHO) guidelines, the treatment criteria have been initiated to all patients with an effort to make more HIV-positive people eligible for therapy [19].

Cervical cancer is potentially preventable and treatable forms of cancer, so that morbidity and mortality could be reduced with early detection and effective management [20, 21]. A number of screening methods are available, such as HPV DNA testing, cytological tests, and visual inspection tests [22–24]. The WHO recommended the need of cervical cancer screening for women aged 30–49 years irrespective of their HIV serostatus and for sexually active girls and women as soon as they are diagnosed as positive for HIV [25]. Unfortunately, many women in developing countries are diagnosed for cervical cancer at late stages of the disease because of poor access to prevention options [26]. Furthermore, there is a lack of both opportunistic and organized population-based screening amongst HIV-positive women due to scarce resources [27].

Ethiopia adopted cervical cancer prevention and control guideline from the WHO and recommend women to get screening for cervical cancer at least every five years following normal results irrespective of HIV status interval to achieve 80% coverage [28, 29]. Currently, both arranged and opportunistic cervical cancer screening are available, and a number of women are expected to be benefited from it [23, 30].

Several fragmented studies have been conducted to assess uptake of cervical cancer screening and associated factors among HIV-positive women in Ethiopia. However, the studies reported greatly wide-ranging and inconsistent findings, and this variation has not been examined systematically. Thus, this systematic review and meta-analysis aims to answer the following questions: What is the magnitude of cervical cancer screening uptake among HIV-positive women in Ethiopia? What are the factors associated with the uptake of cervical cancer screening among HIV-positive women in Ethiopia? Accordingly, the population, intervention, comparator, outcome, study design (PICOS) framework for these questions was as follows: (P) HIV-positive women, (I) uptake of cervical cancer screening, (C) no screening, and (O) uptake of cervical cancer screening and associated factors among HIV-positive women. This study may help for policymakers, programmers, and other concerned bodies to design the best possible screening strategies for cervical cancer that can have a large impact on the women's quality of life and expectancy. Furthermore, the finding of this study may help the health workers to integrate cervical cancer screening services and counseling within their activities, so that the incidence and mortality associated with cervical cancer could be reduced through early diagnosis and treatment.

## 2. Methods

### 2.1. Search Strategy and Information Sources

This systematic review and meta-analysis was prepared and presented according to the Preferred Reporting Items for Systematic Reviews and Meta-Analysis (PRISMA) checklist [31]. A comprehensive search was performed to find potentially relevant articles in the following databases: PubMed/MEDLINE, Scopus, Web of Science, EMBASE, CINAHL, Google Scholar, Science Direct, and Cochrane Library. To find unpublished articles, review of reference lists, input of content experts, and Institutional Digital Library were searched. In addition, the reference lists of articles that were considered relevant to this systematic review and meta-analysis were also searched. The search was carried out based on the following key words: “uptake,” “utilization,” “cervical cancer,” “cervical neoplasm,” “screening,” “early detection,” “determinants,” “associated factors,” “HIV positive,” “HIV seropositivity,” “women,” and “Ethiopia.” For the systematic identification of records, Boolean operators (AND, OR), truncation, and the MeSH terms were used appropriately. The search was conducted from 8th of May to 24th of May, 2020. Finally, identified studies were retrieved and managed using the Endnote X7 software.

### 2.2. Inclusion and Exclusion Criteria

#### 2.2.1. Inclusion Criteria

*(1) Study Area*. Studies conducted only in Ethiopia.

*(2) Study Design*. This review considered all observational study designs (i.e., cross-sectional, case-control, and cohort).

*(3) Population*. Studies involving HIV-positive women and reported the uptake of cervical cancer screening and associated factors among HIV-positive women were eligible for this study.

*(4) Language*. Only studies conducted and reported in English language were considered.

*(5) Publication Condition*. Both published and unpublished articles were included.

#### 2.2.2. Exclusion Criteria

Articles that did not report specific outcomes for uptake of cervical cancer screening among HIV-positive women quantitatively were excluded. In addition, studies that did not report the odds ratio (OR) or binary outcomes were not included to examine factors associated with uptake of cervical cancer screening. Furthermore, studies that were not fully accessible, qualitative studies, reviews, case series, and reports and studies that show trends were excluded.

### 2.3. Study Selection

The studies were selected first based on the relevance of their titles and abstract. Next, full-text articles were retrieved and reviewed to confirm eligibility. The PRISMA flow diagram was used to summarize the study selection processes.

### 2.4. Measurement of Outcome Variables

The primary outcome variable of this study is uptake of cervical cancer screening, which is defined as HIV-positive women who were screened for premalignant cervical lesion at least once in their life time. The second outcome of this study was to identify risk factors associated with uptake of cervical cancer screening. To examine determinants, data were extracted from the primary studies in the form of two-by-two tables, and OR was calculated to determine the association between each of the factors and uptake of cervical cancer screening. The main criterion for selecting variables was how clearly and frequently they were reported in the studies included in the meta-analysis. Accordingly, determinants reported in more than one study and having odds ratio or binary outcomes were included. The determinants included in this review were knowledge of women about cervical cancer (good versus poor), educational status of women (formal education versus no formal education), and perceived susceptibility of developing cervical cancer (high perceived versus low perceived).

### 2.5. Risk of Bias (Quality) Assessment

Each individual article was critically appraised using the Joanna Briggs Institute (JBI) Meta-Analysis of Statistics Assessment and Review Instrument adapted for studies reporting prevalence data, cross-sectional, cohort, and case-control studies [32]. The tool mainly included criteria for inclusion, detail description of study subjects and setting, reliability and validity of study tools, representation of the population and methods of participants' selection, acceptability of case definition, identification of cofounders, strategies dealing with cofounders, validity of measurement to test outcome, and appropriateness of statistical tests. The overall score of risk of bias was categorized according to the number of high risk of bias per study: each question is scored as Yes (1) or No (0), Unclear (0), and Not applicable (0). In this systematic review and meta-analysis, all included studies fulfilled the JBI-recommended quality criteria, which is scored ≥60% [33].

### 2.6. Data Extraction

All necessary data from included articles were extracted using a standardized data extraction format, adapted from the JBI. Data extraction sheet included study characteristics such as authors' name, region, study year, publication year, study design, study setting, sample size, response rate, sampling, uptake of cervical cancer screening (prevalence), and odds ratio or binary outcomes of each identified risk factors.

### 2.7. Data Processing and Analysis

The extracted data were entered into an excel sheet and then imported to STATA, version 14, for analysis. For each original study, the standard error was calculated using the binomial distribution formula. Heterogeneity among reported prevalence of cervical cancer screening was assessed by using the inverse variance (*I*^2^) with Cochran *Q* statistic and a cutoff of 25%, 50%, and 75% were declared as low, moderate, and sever heterogeneity, respectively, with *p* value less than 0.05 [34]. Since heterogeneity was exhibited among studies (*I*^2^ = 96.6%, *p* < 0.001), a random effects model was used to estimate the pooled prevalence of cervical cancer screening uptake among HIV-positive women and was presented using forest plot with 95% confidence interval (CI). For the second outcomes, pooled odds ratios with 95% CI for each factor were used to determine the association between uptake of cervical cancer screening and independent variables.

In addition, evidence of publication bias was assessed using asymmetry of funnel plots and Egger's test with a *p* value of less than 0.05 as a cutoff point to declare the presence of publication bias [35]. The results revealed that no evidence of publication bias was observed.

## 3. Results

### 3.1. Selection of Studies

**Figure 1:**
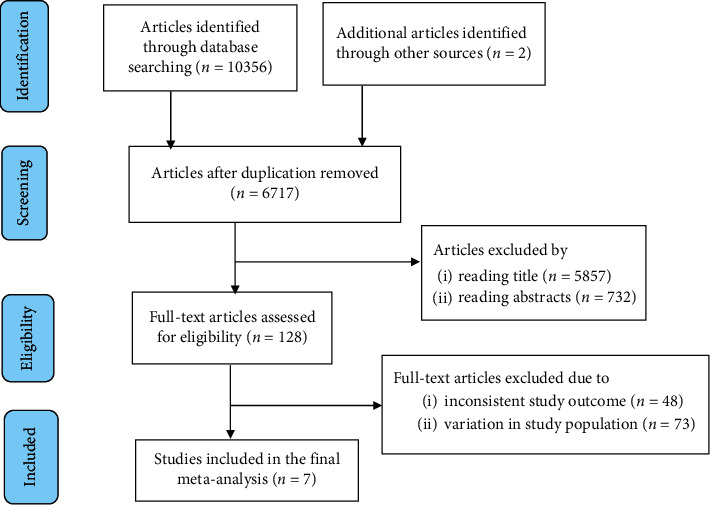
Flow chart of study selection for systematic review and meta-analysis of cervical cancer screening uptake among HIV-positive women in Ethiopia, 2020.

A total of 10358 articles were identified through the electronic searches of different databases. Of which, 3641 articles were removed due to duplication. From the remaining 6717 articles, 6589 articles were excluded after reading of titles and abstracts. The full text of the remaining 128 articles were accessed and assessed for eligibility criteria, further yielding exclusion of 121 articles because their outcomes were not clearly stated and variation in study population. Ultimately, the remaining 7 studies were included in the systematic review and meta-analysis (Figure 1).

### 3.2. Characteristics of Included Studies

All included studies used facility-based cross-sectional study design to estimate uptake of cervical cancer. With the exception of one study that is unpublished then done in 2018 [36], all the included studies were published from 2015 up to 2019. All the studies used interviewer-administered questionnaire, adapted from reviving previous studies, to collect the data, and also they used systematic random sampling method to select study participants. A total of 2822 participants were involved from estimated 2922 HIV-positive women, yielding a response rate of 96.6% with an estimated sample size ranging from 317 [37] up to 594 [38]. All of the included studies had high response rates (>90%). The included studies reported that the prevalence of cervical cancer screening among HIV-positive women ranged from 7.8% [36] to 40.1% [39]. The quality score of included studies indicated that 6 articles were deemed to be of high quality, 1 of moderate quality. Two of the studies included in this review were from Addis Ababa administrative city [38, 40], two were from Amhara Region [37, 41], one was from Southern Nations Nationalities and People's Region (SNNPR) [39], one was from Oromia Region [42], and the remaining one was from Tigray Region [36] (Table 1).

### 3.3. Quality Assessment

The methodological quality of the findings of the included studies was critically evaluated using the Joanna Briggs Institute (JBI) Meta-Analysis of Statistics Assessment and Review Instrument adapted for analytical cross-sectional studies. Majority of the studies (*n* = 6, 85.7%) were determined to be of high quality, that is, a score of ≥7 “yes” out of 8 on the quality scale which is ≥87.5%. Only one study (14.3%) were considered moderate quality, that is, a score of 6 “yes” out of 8 on the quality scale which is 75%. Three studies did not describe the strategies how to dealing with cofounders. Most of the studies (*n* = 6) used standard criteria or objective to measure conditions (Table 2).

### 3.4. Uptake of Cervical Cancer Screening among HIV-Positive Women in Ethiopia

**Figure 2:**
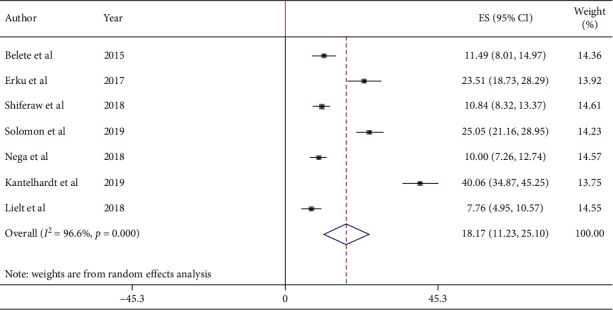
Forest plot of the pooled prevalence of cervical cancer screening uptake among HIV-positive women in Ethiopia, 2020.

Overall, the pooled prevalence of cervical cancer screening uptake among HIV-positive women in Ethiopia was 18.17% (95% CI : 11.23, 25.10). High heterogeneity was exhibited (*I*^2^ = 96.6%; *p* < 0.001) in estimating the pooled prevalence of cervical cancer screening among HIV-positive women. Thus, random effects model was computed to estimating the pooled uptake of cervical cancer screening among HIV-positive women in Ethiopia (Figure 2).

### 3.5. Publication Bias

**Figure 3:**
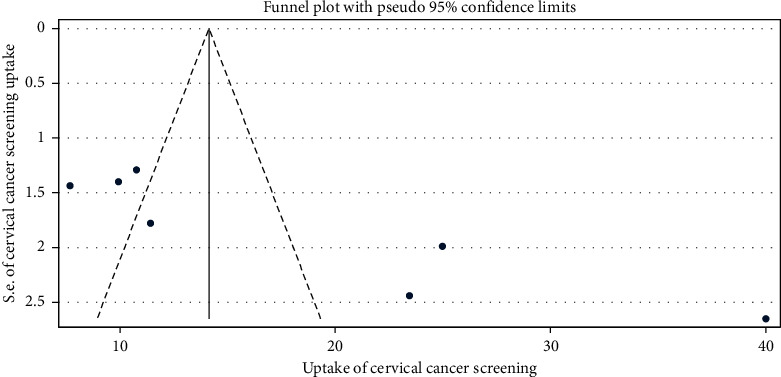
Graphic representation of publication bias using funnel plots of all included studies, 2020.

The result of both funnel plots of precision asymmetry and Egger's test of the intercept revealed the absence of publication bias in the included studies. The result of Egger's test declared that publication bias was not statistically significant(*p*=0.068). In addition, visual inspection of the funnel plots indicated a symmetric distribution of studies (Figure 3).

### 3.6. Factors Associated with Uptake of Cervical Cancer Screening

In this review, some of the factors associated with uptake of cervical cancer screening were pooled quantitatively and some were not due to inconsistent grouping (classification) of the independent variables with respect to the outcome (uptake of cervical cancer screening). Age of women, parity, length of time after diagnosis as HIV positive, and CD4 count were factors reported in more than one primary studies but not included in this meta-analysis because of inconsistent classification in primary studies. Thus, those factors reported in more than one primary studies and had consistent classification (knowledge, educational status, and perceived susceptibility) were included in this meta-analysis.

**Figure 4:**
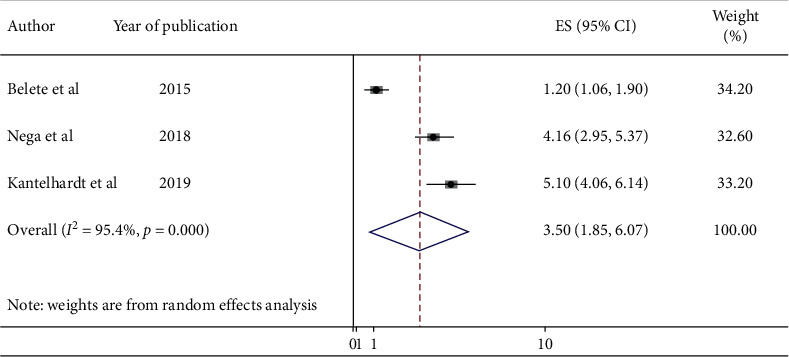
Forest plot showing the pooled odds ratio of the association between educational status and cervical cancer screening uptake among HIV-positive women in Ethiopia, 2020.

Three studies indicated educational status of women has a significant association with uptake of cervical cancer screening. The odd of being screened among HIV-positive women having formal education were 3.50 times (AOR = 3.50; 95% CI : 1.85, 6.07) higher than HIV-positive women who have no formal education (Figure 4).

**Figure 5:**
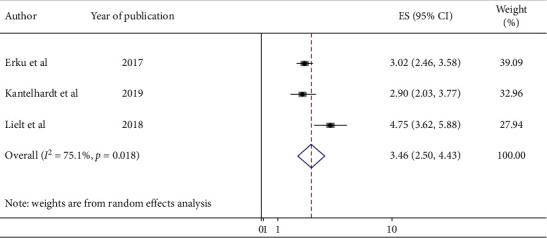
Forest plot showing the pooled odds ratio of the association between knowledge and cervical cancer screening uptake among HIV-positive women in Ethiopia, 2020.

Three studies also indicated that knowledge of women on cervical cancer has a significant association with uptake of cervical cancer screening. HIV-positive women who had good knowledge of cervical cancer were 3.46 times (AOR = 3.26; 95% CI : 2.50, 4.43) more likely to be screened for cervical cancer than those who had poor knowledge (Figure 5).

**Figure 6:**
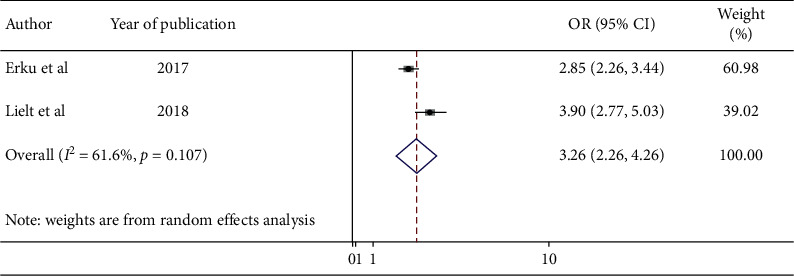
Forest plot showing the pooled odds ratio of the association between perceived susceptibility and cervical cancer screening uptake among HIV-positive women in Ethiopia, 2020.

Furthermore, two studies revealed that perceived susceptibility of women has a significant association with uptake of cervical cancer screening. The odds of cervical cancer screening uptake among HIV-positive women who had high perception on their susceptibility to develop cervical cancer were 3.26 times (AOR = 3.26; 95% CI : 2.26, 4.26) higher than those who had low perception (Figure 6).

## 4. Discussion

The fundamental goal of cervical screening is to reduce the incidence of and subsequent mortality from invasive cervical cancer [43]. Consequently, effective screening intervention and implementation approaches are warranted if the full benefits of screening are to be recognized. Thus, this systematic review and meta-analysis was conducted to estimate the pooled prevalence of cervical cancer screening uptake and its associated factors among HIV-positive women in Ethiopia.

According to this systematic review and meta-analysis, less than one-fifth (18.17%) of HIV-positive women are screened for cervical cancer in Ethiopia, which is much lower than the national target of cervical cancer screening [29]. This finding implies that the need for close monitoring and evaluation of screening service utilization, coverage, and sustainability of a cervical cancer prevention program in Ethiopia. In addition, this finding infers the collaborative effort among different stakeholders to be engaged in advocacy, social mobilization, and dissemination of cervical cancer prevention program activities. Moreover, this study indicates the need of expansion and strengthening screening activities at different levels of the health system.

In the current meta-analysis, the pooled prevalence of cervical cancer screening among HIV-positive women in Ethiopia was 18.17% (95% CI : 11.23, 25.10). Even if there is no similar meta-analysis study conducted on this specific research question, the results of the finding is higher compared with the study done in Nigeria (9%) [44] and Morocco (9%) [45]. This variation might be due to differences in access to reproductive health services, and community sensitization and awareness creation programs on cervical cancer as well as cervical screening service. For instance, in Ethiopia, community mobilization and awareness creation through expansion of urban and rural health extension programs has been put into effect in recent years. Moreover, differences in sociodemographic and economic status of the study participants could be another reason for the reported variations.

However, this finding is lower than the study findings conducted in England 85.7% [46], Canada 58% [47], Catalonia 50.6% [48], Italy 91% [49], and Kenya 46% [50]. The reason for this variation could be due to differences in study participants' level of awareness about cervical cancer and its screening program. In addition, it could be due to variations of countries' promotional policy, enhanced nationwide advocacy, and media concern. For instance, in Canada, screening utilization could be increased as even distribution of screening services centers and universal access to health care is available [51]. Likewise, Kenya has a more robust cervical cancer screening program and other prevention services as well as continuous provision of health education about cervical cancer and screening activities [52]. On the other hand, in Ethiopia, cervical cancer screening activities are not expanded at different levels of the health system, and cervical screening service was initiated just few years back as well as continuous and organized health education and awareness creation programs are not well established [41].

This systematic review and meta-analysis revealed that HIV-positive women having formal education were 3.50 times more likely to be screened for cervical cancer as compared with HIV-positive women who have no formal education. This finding was supported by previous studies [44, 53, 54]. The reason could be the fact that women who are educated have better understanding of the cause, risk factors, prevention mechanism, and treatment of the disease given from healthcare workers, mass media, and different stakeholder. Furthermore, better-educated women are expected to have motivation, confidence, and social inclusion in search for information and health interventions [55].

Knowledge of women on cervical cancer was also another determinant of cervical cancer screening uptake. Accordingly, HIV-positive women who had good knowledge of cervical cancer were 3.46 times more likely to be screened for cervical cancer than those who had poor knowledge. This finding is similar with other previous studies [46, 56, 57]. This finding might be due to the fact that HIV-positive women having good knowledge about the severity and risk factors of cervical cancer would have better health-seeking behavior, so that they can utilize screening service of cervical cancer uptake.

Furthermore, this study indicated that perceived susceptibility of women has a significant association with uptake of cervical cancer screening. The odds of cervical cancer screening uptake among HIV-positive women who had high perception on their susceptibility to develop cervical cancer were 3.26 times higher than those who had low perception. This finding is supported by other studies [56, 58]. This finding is also supported by the study conducted among HIV-infected women in Florida [46], which reported that low perception of susceptibility is associated with low utilization of the screening service. The reason could be the fact that HIV-positive women who perceive themselves as susceptible to develop cervical cancer could be motivated for screening earlier before attaining the disease. Literature suggested that self-vulnerability to illness is an important predictor of early screening and better health-related activities [59].

### 4.1. Limitations of the Study

This systematic review and meta-analysis encountered several limitations: first, the possibility of missing some relevant articles is expected as articles published only in the English language were included and studies could not be accessed in full text were excluded after failing repeated e-mail communication with the author. In addition, the heterogeneity across selected studies was high, but subgroup analysis was not done and other important sources of heterogeneity were not fully addressed. Furthermore, since the selected study was not from all the regions of Ethiopia and minimal in number, the generalizability of the study might not be in full confidence. Furthermore, the pooled odds ratio for all variables associated with cervical cancer screening uptake among HIV-positive women were not examined because some studies did not report the likely predictors or risk factors, and some studies that reported the factors associated with cervical cancer screening uptake were inconsistent. Moreover, generalizability of the finding may be reduced since all the included studies were facility based and cross-sectional.

## 5. Conclusion

This systematic review and meta-analysis found that cervical cancer screening uptake among HIV-positive women in Ethiopia is low compared with the national target of cervical cancer screening coverage. Educational status of women, knowledge of women on cervical cancer, and perceived susceptibility to develop cervical cancer were the identified factors associated with cervical cancer screening uptake among HIV-positive women in Ethiopia. The findings of this study highlight the need to improving the existing national strategies of cervical cancer screening so as to strengthen reproductive health education, promotion, and community sensitization in addition to providing screening services. In addition, policymakers, programmers, implementers, and different stakeholders working on cancer and HIV/AIDS should provide a customized health promotion and awareness creation interventions among HIV-infected women. Furthermore, cervical cancer screening service should be integrated to the routine care and treatment, so that HIV-positive women can get counseling services from health care workers in every clinical contact. Moreover, there should be consistent monitoring and evaluation on the detection of cervical cancer and prevention activities.

## Figures and Tables

**Table 1:** Descriptive summary of primary studies included in the systematic review and meta-analysis of cervical cancer screening uptake among HIV-positive women in Ethiopia, 2020.

First author	Year	Region	Study area	Study design	Study population	Sample size	Number of participants	Response rate (%)	Screening uptake (%)
Belete et al. [40]	2015	Addis Ababa	Addis Ababa	Facility-based cross-sectional	HIV-positive women above age 17	333	322	96.7	11.5
Erku et al. [37]	2017	Amhara	Gondar Hospital	Facility-based cross-sectional	HIV-positive women above age 17	317	302	95.3	23.5
Shiferaw et al. [38]	2018	Addis Ababa	Addis Ababa	Facility-based cross-sectional	HIV-positive women of age 21–64	594	581	97.8	10.8
Solomon et al. [42]	2019	Oromia	Bishoftu	Facility-based cross-sectional	HIV-positive women above age 18	475	467	98.3	25.1
Nega et al. [41]	2018	Amhara	Gondar Hospital	Facility-based cross-sectional	HIV-positive women above age 18	496	460	92.7	10.0
Kantelhardt et al. [39]	2019	SNNPR	Hawassa town	Facility-based cross-sectional	All HIV-positive women	350	342	97.7	40.1
Lielt et al. [36]	2018	Tigray	Alamata Hospital	Facility-based cross-sectional	HIV-positive women and unknown HIV status	357	348	98.3	7.8

**Table 2:** Joanna Briggs Institute Critical Appraisal Checklist for analytical cross-sectional studies, 2020.

Studies	Clear criteria for inclusion	Detail description of study subject and setting	Reliability and validity of study tools	Used standard criteria or objective	Identify cofounding factor	Strategy dealing with cofounders	Outcome measured in a valid way	Appropriate statistical analysis used	Overall score (%)
Belete et al. [40]	Yes	Yes	Yes	Yes	Yes	Yes	Yes	Yes	100
Erku et al. [37]	Yes	Yes	Yes	Yes	Yes	No	Yes	Yes	87.5
Shiferaw et al. [38]	Yes	Yes	Yes	Yes	Yes	No	Yes	Yes	87.5
Solomon et al. [42]	Yes	Yes	Yes	Yes	Yes0	Yes	Yes	Yes	100
Nega et al. [41]	Yes	Yes	Yes	Yes	Yes	Yes	Yes	Yes	100
Kantelhardt et al. [39]	Yes	Yes	Yes	Yes	Yes	No	Yes	Yes	87.5
Lielt et al. [36]	Yes	No	Yes	Unclear	Yes	Yes	Yes	Yes	75

## Data Availability

All relevant data are included within the article.

## Abbreviations

AOR: Adjusted odd ratio
CI: Confidence intervals
HIV: Human immunodeficiency virus
HPV: Human papillomavirus
OR: Odds ratio
WHO: World Health Organization.

## References

[1] Tabrizi S. N., Brotherton J. M. L., Kaldor J. M. (2012). Fall in human papillomavirus prevalence following a national vaccination program. *Journal of Infectious Diseases*.

[2] Bateson J., Shin H., Bray F., Forman D., Mathers C., Parkin D. (2012). *GLOBOCAN 2008 V1. 2, Cancer Incidence and Mortality Worldwide: IARC CancerBase No. 10*.

[3] Siegel R., Jemal A. (2020). *Cancer Facts & Figures 2015*.

[4] Crosbie E. J., Einstein M. H., Franceschi S., Kitchener H. C. (2013). Human papillomavirus and cervical cancer. *The Lancet*.

[5] World Health Organization (2016). *Human Papillomavirus (HPV) and Cervical Cancer*.

[6] Fitzmaurice C., Fitzmaurice C., Dicker D. (2015). The global burden of cancer 2013. *JAMA Oncology*.

[7] Naghavi MacIntyre E. H. (2014). Related cancers, fact sheet 2014. ICO Information centre on HPV and cancer. https://hpvcentre.net/statistics/reports/ETH_FS.pdf.

[8] McDonald A. C., Tergas A. I., Kuhn L., Denny L., Wright T. C. (2014). Distribution of human papillomavirus genotypes among HIV-positive and HIV-negative women in Cape Town, South Africa. *Frontiers in Oncology*.

[9] Denslow S. A., Rositch A. F., Firnhaber C., Ting J., Smith J. S. (2014). Incidence and progression of cervical lesions in women with HIV: a systematic global review. *International Journal of STD & AIDS*.

[10] Wild C. P., Stewart B. W., Wild C. (2014). *World Cancer Report 2014*.

[11] Gedefaw A., Astatkie A., Tessema G. A. (2013). The prevalence of precancerous cervical cancer lesion among HIV-infected women in southern Ethiopia: a cross-sectional study. *PLoS One*.

[12] Bratcher L. F., Sahasrabuddhe V. V. (2010). RTehvieew impact of antiretroviral therapy on HPV andcervical intraepithelial neoplasia: current evidence and directions for future research. *Infectious Agents and Cancer*.

[13] Clifford G. M., Franceschi S., Keiser O. (2016). Immunodeficiency and the risk of cervical intraepithelial neoplasia 2/3 and cervical cancer: a nested case-control study in the Swiss HIV cohort study. *International Journal of Cancer*.

[14] Levi C. E., O’Bryan G., Tchago F. E., Nangue C., Bekoule P. S., Kollo B. (2016). Integrating cervical cancer screening with HIV care in Cameroon: comparative risk analysis of cervical disease in HIV-infected women receiving antiretroviral therapy to women in the general population. *PLoS One*.

[15] Tanon A., Jaquet A., Ekouevi D. K. (2012). The spectrum of cancers in West Africa: associations with human immunodeficiency virus. *PLoS One*.

[16] Sichanh C., Fabrice Q., Chanthavilay P. (2014). Knowledge, awareness and attitudes about cervical cancer among women attending or not an HIV treatment center in Lao PDR. *BMC Cancer*.

[17] Staff P. O. (2015). Correction: retention in care of adult HIV patients initiating antiretroviral therapy in Tigray, Ethiopia: a prospective observational cohort study. *PLoS One*.

[18] Yaya I., Landoh D. E., Saka B. (2014). Predictors of adherence to antiretroviral therapy among people living with HIV and AIDS at the regional hospital of Sokodé. *Togo. BMC Public Health*.

[19] Antiretroviral TS (2015). Guideline on when to start antiretroviral therapy and on pre-exposure prophylaxis for HIV. https://appswhoint/iris/bitstream/handle/10665/186275/9789241509565_engpdf?sequence=1&ua=1.

[20] Kleinhaus A. L. (1975). Electrophysiological actions of convulsants and anticonvulsants on neurons of the leech subesophageal ganglion. *Comparative Biochemistry and Physiology C: Comparative Pharmacology*.

[21] Finocchario-Kessler S., Wexler C., Maloba M., Mabachi N., Ndikum-Moffor F., Bukusi E. (2016). Cervical cancer prevention and treatment research in Africa: a systematic review from a public health perspective. *BMC Women’s Health*.

[22] Fallala M. S., Mash R. (2015). Cervical cancer screening: safety, acceptability, and feasibility of a single-visit approach in Bulawayo, Zimbabwe. *African Journal of Primary Health Care & Family Medicine*.

[23] Lim J. N., Ojo A. A. (2017). Barriers to utilisation of cervical cancer screening in sub Sahara Africa: a systematic review. *European Journal of Cancer Care*.

[24] Richter K. (2015). *Implementation of HPV Vaccination in South Africa*.

[25] World Health Organization (2013). *WHO Guidelines for Screening and Treatment of Precancerous Lesions for Cervical Cancer Prevention*.

[26] McFarland D. M., Gueldner S. M., Mogobe K. D. (2016). Integrated review of barriers to cervical cancer screening in sub-saharan Africa. *Journal of Nursing Scholarship*.

[27] Health WHOR (2014). *Comprehensive Cervical Cancer Control: A Guide to Essential Practice:*.

[28] Memirie S. T., Habtemariam M. K., Asefa M. (2018). Estimates of cancer incidence in Ethiopia in 2015 using population-based registry data. *Journal of Global Oncology*.

[29] Abraha E. (2015). Guideline for cervical cancer prevention and control in Ethiopia. https://www.iccp-portal.org/system/files/plans/Guideline%20Eth%20Final.pdf.

[30] Getahun F., Addissie A., Negash S., Gebremichael G. (2019). Assessment of cervical cancer services and cervical cancer related knowledge of health service providers in public health facilities in Addis Ababa, Ethiopia. *BMC Research Notes*.

[31] Moher D., Liberati A., Tetzlaff J., Altman D. G., Group P. (2009). Preferred reporting items for systematic reviews and meta-analyses: the PRISMA statement. *PLoS Medicine*.

[32] Briggs I. J. (2016). JBI critical appraisal checklist for analytical cross sectional studies.

[33] Porritt K., Gomersall J., Lockwood C. (2014). JBIʼs systematic reviews. *American Journal of Nursing*.

[34] Rücker G., Schwarzer G., Carpenter J. R., Schumacher M. (2008). Undue reliance on I 2 in assessing heterogeneity may mislead. *BMC Medical Research Methodology*.

[35] Egger M., Smith G. D., Schneider M., Minder C. (1997). Bias in meta-analysis detected by a simple, graphical test. *BMJ*.

[36] Lieltal G. (2018). Cervical cancer screening service utilization and associated factors among HIV positive and women with unknown status in Alamata generalized hospital, Tigray, Ethiopia 2018: Comparative cross sectional study.

[37] Erku D. A., Netere A. K., Mersha A. G., Abebe S. A., Mekuria A. B., Belachew S. A. (2017). Comprehensive knowledge and uptake of cervical cancer screening is low among women living with HIV/AIDS in Northwest Ethiopia. *Gynecologic Oncology Research and Practice*.

[38] Shiferaw S., Addissie A., Gizaw M. (2018). Knowledge about cervical cancer and barriers toward cervical cancer screening among HIV-positive women attending public health centers in Addis Ababa city, Ethiopia. *Cancer Medicine*.

[39] Kantelhardt A. A., Astawesegn F. H., Eshetu B. (2019). Cervical cancer screening service utilization and associated factors among HIV-positive women attending adult ART clinic in public health facilities, Hawassa town, Ethiopia: a cross-sectional study. *BMC Health Services Research*.

[40] Belete N., Tsige Y., Mellie H. (2015). Willingness and acceptability of cervical cancer screening among women living with HIV/AIDS in Addis Ababa, Ethiopia: a cross sectional study. *Gynecologic Oncology Research and Practice*.

[41] Nega A. D., Woldetsadik M. A., Gelagay A. A. (2018). Low uptake of cervical cancer screening among HIV-positive women in Gondar University referral hospital, Northwest Ethiopia: cross-sectional study design. *BMC Women’s Health*.

[42] Solomon K., Tamire M., Kaba M. (2019). Predictors of cervical cancer screening practice among HIV-positive women attending adult anti-retroviral treatment clinics in Bishoftu town, Ethiopia: the application of a health belief model. *BMC Cancer*.

[43] Peirson L., Fitzpatrick-Lewis D., Ciliska D., Warren R. (2013). Screening for cervical cancer: a systematic review and meta-analysis. *Systematic Reviews*.

[44] Ezechi O. C., Gab-Okafor C. V., Ostergren P. O., Pettersson K. O. (2013). Willingness and acceptability of cervical cancer screening among HIV-positive Nigerian women. *BMC Public Health*.

[45] Belglaiaa E., Souho T., Badaoui L. (2018). Awareness of cervical cancer among women attending an HIV treatment centre: a cross-sectional study from Morocco. *BMJ Open*.

[46] Ogunwale A. N., Coleman M. A., Sangi-Haghpeykar H. (2016). Assessment of factors impacting cervical cancer screening among low-income women living with HIV-AIDS. *AIDS Care*.

[47] Anderson P., Kendall C., Touchie C., Pottie K., Angel J. B., Jaffey J. (2010). Cervical cancer screening among HIV-positive women. Retrospective cohort study from a tertiary care HIV clinic. *Canadian family physician Medecin de famille canadien*.

[48] Stuardo V. (2013). Low prevalence of cervical cancer screening among HIV-positive women in Catalonia (Spain). *Journal of AIDS and Clinical Research*.

[49] Dal Maso L., Franceschi S., Lise M. (2010). Self-reported history of Pap-smear in HIV-positive women in Northern Italy: a cross-sectional study. *BMC Cancer*.

[50] Njuguna E., Ilovi S., Muiruri P., Mutai K., Kinuthia J., Njoroge P. (2017). Factors influencing cervical cancer screening in a Kenyan health facility: a mixed qualitative and quantitative study. *International Journal of Reproduction, Contraception, Obstetrics and Gynecology*.

[51] Maar M., Wakewich P., Wood B. (2016). Strategies for increasing cervical cancer screening amongst first nations communities in Northwest Ontario, Canada. *Health Care for Women International*.

[52] Ogilvie J. I., Njoroge B., Huchko M. J. (2015). Cervical cancer screening knowledge and behavior among women attending an urban hiv clinic in Western Kenya. *Journal of Cancer Education*.

[53] Nene B., Jayant K., Arrossi S. (2007). Determinants of women’s participation in cervical cancer screening trail, Maharashtra, India. *Bulletin of the World Health Organization*.

[54] Ebu N. I. (2018). Socio-demographic characteristics influencing cervical cancer screening intention of HIV-positive women in the central region of Ghana. *Gynecologic Oncology Research and Practice*.

[55] Adanu R., Seffah J., Duda R., Darko R., Hill A., Anarfi J. (2010). Clinic visits and cervical cancer screening in Accra. *Ghana Medical Journal*.

[56] Oba S., Toyoshima M., Ogata H. (2017). Association of cervical cancer screening with knowledge of risk factors, access to health related information, health profiles, and health competence beliefs among community-dwelling women in Japan. *Asian Pacific Journal of Cancer Prevention :APJCP*.

[57] Leung S., Leung I. (2010). Cervical cancer screening: knowledge, health perception and attendance rate among Hong Kong Chinese women. *International Journal of Women’s Health*.

[58] Morema E. N., Atieli H. E., Onyango R. O., Omondi J. H., Ouma C. (2014). Determinants of cervical screening services uptake among 18–49 year old women seeking services at the Jaramogi Oginga Odinga Teaching and Referral Hospital, Kisumu, Kenya. *BMC Health Services Research*.

[59] Ndikom C. M., Ofi B. A. (2012). Awareness, perception and factors affecting utilization of cervical cancer screening services among women in Ibadan, Nigeria: a qualitative study. *Reproductive Health*.

